# MitImpact 3: modeling the residue interaction network of the Respiratory Chain subunits

**DOI:** 10.1093/nar/gkaa1032

**Published:** 2020-12-09

**Authors:** Stefano Castellana, Tommaso Biagini, Francesco Petrizzelli, Luca Parca, Noemi Panzironi, Viviana Caputo, Angelo Luigi Vescovi, Massimo Carella, Tommaso Mazza

**Affiliations:** Laboratory of Bioinformatics, IRCCS Casa Sollievo della Sofferenza, San Giovanni Rotondo (FG), 71013, Italy; Laboratory of Bioinformatics, IRCCS Casa Sollievo della Sofferenza, San Giovanni Rotondo (FG), 71013, Italy; Laboratory of Bioinformatics, IRCCS Casa Sollievo della Sofferenza, San Giovanni Rotondo (FG), 71013, Italy; Department of Experimental Medicine, Sapienza University of Rome, Rome 00161, Italy; Laboratory of Bioinformatics, IRCCS Casa Sollievo della Sofferenza, San Giovanni Rotondo (FG), 71013, Italy; Department of Experimental Medicine, Sapienza University of Rome, Rome 00161, Italy; Department of Experimental Medicine, Sapienza University of Rome, Rome 00161, Italy; ISBReMIT Institute for Stem Cell Biology, Regenerative Medicine and Innovative Therapies, IRCSS Casa Sollievo della Sofferenza, San Giovanni Rotondo (FG), 71013, Italy; Laboratory of Medical Genetics, IRCCS Casa Sollievo della Sofferenza, San Giovanni Rotondo (FG) 71013, Italy; Laboratory of Bioinformatics, IRCCS Casa Sollievo della Sofferenza, San Giovanni Rotondo (FG), 71013, Italy

## Abstract

Numerous lines of evidence have shown that the interaction between the nuclear and mitochondrial genomes ensures the efficient functioning of the OXPHOS complexes, with substantial implications in bioenergetics, adaptation, and disease. Their interaction is a fascinating and complex trait of the eukaryotic cell that MitImpact explores with its third major release. MitImpact expands its collection of genomic, clinical, and functional annotations of all non-synonymous substitutions of the human mitochondrial genome with new information on putative Compensated Pathogenic Deviations and co-varying amino acid sites of the Respiratory Chain subunits. It further provides evidence of energetic and structural residue compensation by techniques of molecular dynamics simulation. MitImpact is freely accessible at http://mitimpact.css-mendel.it.

## INTRODUCTION

The genetics of the mitochondria received a high impulse approximately thirty years ago when a missense variant in the mitochondrial MT-ND4 gene was discovered to cause Leber's Optic Neuropathy (LHON, MIM #535000) (1). Since then, several defects in mito-nuclear genes encoding structural proteins or proteins involved in mitochondrial functions were associated with mitochondrial disorders (2–4) and complex multifactorial diseases (5,6).

Around 1500 proteins appear to be necessary for the mitochondrial biogenesis and metabolism (2,7); the human mitochondrial genome (mtDNA) encodes only 13 of them. Then, despite their differences in terms of size, structure, complexity, and transmission mode, mitochondrial and nuclear genomes do necessarily interact, mainly involving the Respiratory Chain (RC) subunits (8–11), with substantial implications in bioenergetics, adaptation, and disease (12–14). These subunits are among the most complex components of the cellular proteome, which are continuously being investigated from structural (15–18), evolutionary (14), and genetic (2) perspectives.

Variations on mitochondrial proteins associate with a broad range of phenotypes that differ in terms of age at onset, single/multi-organ involvement and inheritance pattern (2). To date, hundreds of mutations in the nuclear and mitochondrial genomes were associated with mitochondrial disorders, although the diagnostic rate ranged only from 20% to 60% in several large cohorts (19–22). It could be assumed that a portion of the undiagnosed cases and cases with variable phenotypes might be caused by composites of underestimated interacting variants of uncertain significance.

Interaction is indeed a fundamental property of all biological systems, which is currently under active study (14,23–25). The evolution, structure, and function of a protein depend on the transient and stable interactions among residues of the same protein or interacting proteins. While more research was devoted to studying the epistasis of nuclear variants (26–29), it is important to notice here that at least 4–5% of all nuclear-encoded pathogenic amino acid substitutions are present as fixed residues within one or more mammalian species. The fact that such *Compensated Pathogenic Deviations* (CPDs) (30) were found in supposedly healthy, non-human species as reference amino acids implies that modulatory mechanisms must co-exist and that this should equally hold for nuclear and mitochondrial proteins.

Numerous lines of evidence showed that the nuclear and mitochondrial genomes need to co-evolve to ensure the efficient functioning of the OXPHOS complexes (14,31–33). The way this happens is fascinating and complicated since it depends on their structure, size, complexity, evolution and inheritance patterns (34,35). However, its comprehension is a critical goal since it would explain much of the mutation penetrance and disease phenotype variability.

The vast availability of genomics data, computational tools, and specialized databases contributed significantly to achieving these findings. Since its release in 2015, MitImpact (36) is one piece of this toolbox, when it first provided the most extensive set of pre-computed pathogenicity predictions for all possible non-synonymous variants of the mtDNA. It quickly became the reference resource to this regard, continuously expanding its content with the predictions of several third-party algorithms, and then including, two years later, the pathogenicity assessments of the best performing meta-predictor, APOGEE (37).

The third and current milestone of MitImpact paves the way to the study of a fascinating and complex trait of the eukaryotic cell: the interaction between amino acids of mitochondrial and nuclear proteins (38,39), by reviewing the interactions that may occur between the amino acids encoded by the mitochondrial genome. Although being complicated, studying these interactions is, in fact, possible using systematic protein-protein interaction screening (40), at the cost of expensive and time-consuming assays. Even targeted modification of the mitochondrial DNA has proven to be overly complicated *in-vitro* (41,42), although recent studies have introduced new gene-editing possibilities (43). Computational algorithms, as well, have faced numerous issues and limitations, including the reduced availability of genome sequences of species that are not economically relevant and of resolved protein structures.

In such a complex field of research, MitImpact expands its knowledge base with new information on protein co-variation, structural consequences of variants at co-varying sites, compensated pathogenic deviations, and with speculations on possible structural and evolutionary compensatory mechanisms.

## MATERIALS AND METHODS

**Figure 1.**
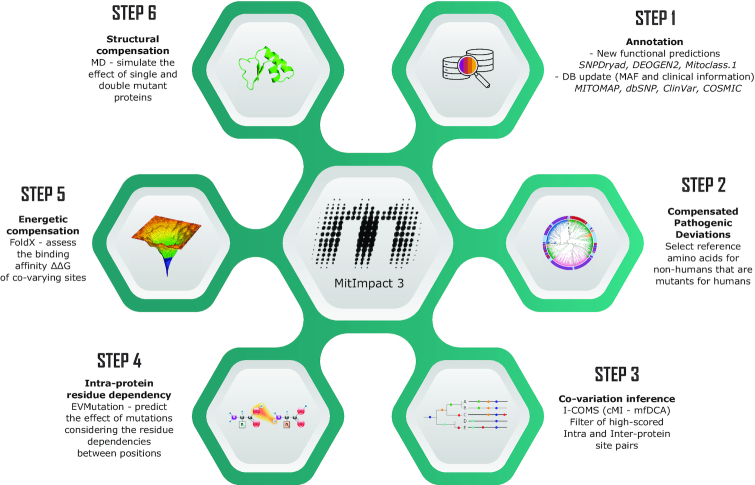
Content schema of MitImpact 3.

In this third major release (Figure 1), MitImpact updates its set of annotations with new content concerning dbSNP v153 (44), ClinVar v153 (45), COSMIC v90 (46) and MITOMAP (February 2020 release) (47), and adds the pathogenicity predictions of three new software packages: SNPDryad (48), DEOGEN2 (49), and MitoClass.1 (50).

CPDs (51,52) were inferred as described in Supplementary File S1B. In brief, the reference protein sequences of the 13 mitochondrial subunits were downloaded from NCBI Organelle (53), restricting the search to the Mammalia clade. The homologous sequences were aligned using ClustalOmega v1.2 (54). Sequences with >50% dissimilarity with the human sequences were discarded. The dissimilarity threshold was set to 30% for MT-ATP8 only. All human missense pathogenic variants were then retrieved from MITOMAP and ClinVar, and localized in the 13 ortholog protein alignments. We considered CPDs the variants that were reference residues in at least one non-human sequence, where at least seven out of the ten surroundings residues, five upstream and five downstream, were identical with the human sequence. This algorithm was inspired by the work of Azevedo and colleagues (52). The final set of CPDs has been made available in MitImpact together with the specification of the species names and NCBI reference sequence identifiers.

We then used I-COMS to study the co-variation of mitochondrial residues (55). The analysis was conducted with two algorithms implemented in I-COMS, cMI (56) and EVFold-mfDCA (57), and was restricted to the orthologs belonging to the Mammalian clade (NCBI37 Taxon ID: 40674). Interactions were considered *inter-protein* whenever residues were located in different proteins of Complex I (twenty-one pairwise analyses), Complex IV (MT-CO1 + MT-CO2, MT-CO2 + MT-CO3, MT-CO1 + MT-CO3) or Complex V (MT-ATP6 + MT-ATP8), and *intra-protein* when both residues were located in the same protein (thirteen pairs), and then not in MT-ND1, which was concatenated by default to all proteins. From the TOP500 inter-protein pairs, in terms of cMI or mfDCA scores, we retained only the pairs whose residues did not fall in the same concatenated protein. From the TOP500 intra-protein pairs, we considered only the pairs where neither residue fell in the MT-ND1 concatenated sequence. Intra-protein interactions were assessed through EVMutation (58) also. We considered the TOP500 scoring pairs, where the higher a score, the stronger was the association between two residues (further details in Supplementary File S1A).

Inter- and intra-protein relationships between co-varying variants were also investigated from an energetic point of view. Atomic coordinates of all human mtDNA-encoded RC proteins, except MT-ATP8, were obtained by homology modeling using the SWISS-MODEL server (59). The MatchMaker extension of UCSF Chimera (60) was used to model the interactions between proteins by superimposing the relative *Bos taurus* structures, the evolutionary closest *Mammalia* whose proteins were available in the Protein Data Bank at the time of this analysis. We performed a minimization step conducted into the membrane environment to refine the interface regions and reduce local errors, such as clashes and Ramachandran outliers. Then, FoldX 4.0 (61) was employed to calculate the free-energy changes upon mutation of the residues lying at the interaction interface (average ΔΔ*G* of 10 replicas) and, consequently, assess the protein stability and protein–protein interaction energy alteration. Alternative amino acids that caused a ΔΔ*G* to exceed the suggested cutoffs (±0.61 kcal/mol) for the single mutant were tagged as disruptive (62). Pairs of mutants with ΔΔG conservatively close to zero (<±0.1 kcal/mol) were considered structurally compensative.

In parallel, we selected the putatively co-varying pairs of residues, where at least one of the pairs was reported as pathogenic in MITOMAP, and both were located in the interaction interface of proteins belonging to the same RC complex. We mapped these residues onto the 3D structure of the human RC I transmembrane arm and investigated the interacting properties of the wild-type complex and the single and double-mutated complexes for all the possible amino acids combinations of each co-varying pair through 50 ns-long molecular dynamics simulations performed using AMBER 18 (63), as described in (64,65). The root-mean-square deviation (RMSD), root mean square fluctuation (RMSF), binding energy components and per-residue hydrogen bonds were measured on the simulated trajectories using GROMACS 4.5 (66) and the *g_mmpbsa* package (67). The essential protein motions' collective coordinates were inferred by principal component analysis of the atomic fluctuations. The 3D movies of the molecular dynamics simulations were obtained using the *g_anaeig* GROMACS tool.

## RESULTS

### Annotations of individual variants

MitImpact 3 stores functional assessments of sixteen predictors, seven meta-predictors, and four cancer-specific predictors for 24.190 amino acid variations in the 13 protein-coding genes of the *Homo sapiens* mitochondrion (NC_012920.1).

*CPD*. Forty-one of these variants were classified as CPDs (Supplementary Data S2) following the workflow described in Supplementary File S1B, where six had already been described in two previous screenings (Table 1).

**Table 1.** Clinically significant amino acid variants in humans that have been identified in mammalian ortholog proteins by this and other published works. Details in Supplementary Data S2

MitImpact ID	Protein variant	Species	Ref.
MI.3124	MT-CO1:p.L196I	*Glis glis* (Rodentia)	(14)
MI.8247	MT-CO3:p.F251L	Many Primates (genus: *Cercocebus, Macaca, Cercopithecus*)	(14)
MI.9927	MT-CYB:p.G251S	Koala, many Primates (genus: e.g., *Pongo, Colobus, Trachypithecus*)	(14)
MI.11461	MT-ND1:p.V113A	*Galeopterus variegatus, Chlorocebus pygerythrus, Chlorocebus cynosures* (Primates)	(68)
MI.7210	MT-CO2:p.V91A	Several Primates and Cetaceans	(68)
MI.23991	MT-ND6:p.I33V	Many Monotrems and Primates	(68)

Only four out of the 41 CPDs were found in at least 10% of the screened mammalian orthologs (Supplementary Data S2), with the alternative residue (Isoleucine) in position 39 in MT-ND1 (MI.10998), which is reported in MITOMAP to be associated with maternally-inherited diabetes and deafness (MIM #520000), resulting in being the reference amino acid in 360/674 MT-ND1 orthologs (53.4%).

### Residue interaction networks

#### Residue co-variation

The total number of inter-protein co-varying pairs of sites was 7.017 (Supplementary Data S3, worksheet ‘cov inter’), 4.285 were found with the cMI method and 2.732 with mfDCA. Forty-seven pairs were in common between the two methods (first 47 rows of the ‘cov common’ worksheet). The intra-protein co-variation analysis generated 6.468 pairs (‘cov intra’), with 3.038 cMI, 3.430 mfDCA and 339 shared pairs (‘cov common’). Given the low congruence of the two analyses (details in Supplementary File S1A), we opted to consider the union of the results of the two methods (i.e. 4.285 and 2.732 inter- and 3.038 and 3.430 intra-protein pairs) for the following energetic analysis steps.

#### Energetically compensatory residues

The co-varying sites were then studied energetically. All the pairs encompassing residues that compensate for the energetic destabilization caused by the other members of the pairs were split into inter-protein (*N* = 256; Supplementary Data S3, ‘comp inter’), intra-protein (*N* = 3.039; ‘comp intra’) and interface-protein (*N* = 219; ‘comp interface’). This last subclass contains double mutants at the interaction interface between RC subunits, including nuclear proteins. An example case study where the energetic destabilization caused by a variant in a gene (i.e. S45P in ND3) can or cannot be restored by a variant in another gene (i.e. N126S and A64P in ND1, respectively) is described in Supplementary File S1C.

#### Structurally compensatory residues

**Figure 2.**
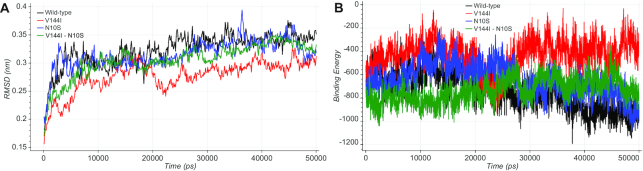
(**A**) The RMSD plot shows how the V144I mutant in MT-ND1 (in red) exhibited lower RMSD values during the simulation time with respect to the wild-type protein complex (in black). N10S in MT-ND3 (in blue) and the double mutant V144I:N10S (in green) showed comparable RMSD values with the wild-type. (**B**) The binding energy plot showed how the V144I variant (in red) causes an alteration of the binding energy values during the entire simulation time compared with the wild-type, while the simultaneous presence of both variants in the MT-ND1:MT-ND3 interface (green) resembles the wild-type condition (black).

The co-varying amino-acid site pairs containing at least one clinically relevant residue were further analyzed by molecular dynamics simulation. Comparing the wild-type protein complexes with the single or double mutants in terms of RMSD, RMSF, binding energy, essential dynamics and the number of hydrogen bonds established or abolished during the entire simulations, we found some cases where the effects of pathogenic alleles have chances to be compensated by co-occurring alleles. For example, the MT-ND3 S45P variant, which is associated with the Leigh syndrome (MIM #256000), is predicted by the previous energetic analysis to be stabilized by three co-occurring other residues located in position 126 of the interacting MT-ND3 protein. Similarly, the MT-ND1 V144I variant is associated with Leber hereditary optic neuropathy (LHON, MIM #535000). The analysis of the molecular dynamics trajectories of this mutant combined with the N10S variant on the interacting MT-ND3 protein showed how the co-occurrence of both mutations could partially restore the fundamental dynamical properties, such as stability and binding affinity (Figure 2). Further details on this were given in **Case study 2: rs201513497** available from the website. Together with 50 other pairs, the results of all MD analyses were stored and made interactively available in a dedicated section of MitImpact.

## DISCUSSION

MitImpact 3 updates its knowledge base and becomes the largest collection of functional predictions of mitochondrial non-synonymous variants. Among all available sixteen pathogenicity predictors, seven meta-predictors, four cancer-specific predictors, our APOGEE (37) stands out for being the reference tool for assessing the pathogenicity of non-synonymous variants in molecular diagnosis (69). However, the most significant update regards the introduction of a new set of putative CPDs, the calculation of the amino acid sites that significantly co-vary within the RC subunits, and the assessment of the energetic and structural compensation caused by pairs of co-varying residues using techniques of molecular dynamics simulation.

These techniques have limitations, as standard procedures to benchmark their results do not exist. The immediate consequence is that different computational methods can yield incongruent results to an extent that is not currently accountable. Caution must also be used with the analyses based on molecular dynamics simulation. These yield results that are indeed more accurate and detailed than those of software predictors, but under the strong hypothesis that the causes of a variant's pathogenic character are ascribed to structural alterations (Supplementary File S1A).

Nevertheless, MitImpact 3 is one of the first specialized bioinformatics resources that adds information on binary epistatic events and their consequences to classical variant annotations and functional predictions, thereby disclosing sites that significantly co-vary through the mammalian phylogeny. Moreover, it stores the results of a great computational effort, which consisted of studying hundreds of co-varying amino acid pairs from energetic and molecular dynamics points of view. The aim of this study, which is still in progress, is that of assessing the structural consequences of known pathogenic variants and pinpointing any eventual compensatory effects. Within the frame of this task, we will create a submission form on the website where interested users can request to analyze energetically and structurally their sets of variants searching for epistatic interactions.

Future developments will deal with the low congruence of methods to assess the co-variation of RC subunits residues. We will use other algorithms and will define a more reliable consensus set of results. We have also planned to release an updated version of APOGEE (37), which will be trained on an updated and finely curated set of known variants. In particular, APOGEE will aggregate other packages' predictions as in version 1 together with co-evolution information from EVMutation, cMI, mfDCA, and ΔΔG folding energy.

MitImpact has been supporting molecular and computational biologists, geneticists, and medical doctors for five years in conducting their work, and will keep doing it freely.

## DATA AVAILABILITY

MitImpact is freely available at http://mitimpact.css-mendel.it. Raw I-COMS score matrices and protein alignments are available at https://mitimpact.css-mendel.it/static/supporting_data/ICOMS_mitimpact3.rar.

Atomic coordinates and structure factors of the human RC I transmembrane arm have been retrieved from the PDB with the following accession number 5xtc. Energetic compensation was calculated on smaller bovine models retrieved from PDB with the following accession numbers: 1be3, 1bgy, 1bmf, 1cow, 1e1q, 1e1r, 1e79, 1efr, 1h8e, 1h8h, 1l0l, 1l0n, 1mab, 1nbm, 1ntk, 1ntm, 1ntz, 1nu1, 1occ, 1oco, 1ocr, 1ocz, 1ohh, 1pp9, 1ppj, 1qcr, 1qo1, 1sqb, 1sqp, 1sqq, 1sqv, 1sqx, 1v54, 1v55, 1w0j, 1w0k, 2a06, 2bcc, 2ck3, 2cly, 2dyr, 2dys, 2eij, 2eik, 2eil, 2eim, 2ein, 2f43, 2fyu, 2jdi, 2jiz, 2jj1, 2jj2, 2occ, 2v7q, 2w6e, 2w6f, 2w6g, 2w6h, 2w6i, 2w6j, 2wss, 2xnd, 2y69, 2ybb, 2zxw, 3abk, 3abl, 3abm, 3ag1, 3ag2, 3ag3, 3ag4, 3asn, 3aso, 3bcc, 3wg7, 3x2q, 4asu, 4b2q, 4d6t, 4d6u, 4tsf, 4tt3, 4yxw, 4z1m, 5ara, 5are, 5arh, 5ari, 5b1a, 5b1b, 5b3s, 5fij, 5fik, 5fil, 5gpn, 5iy5, 5klv, 5lc5, 5ldw, 5ldx, 5lnk, 5luf, 5nmi, 5w97, 5wau, 5x19, 5x1b, 5x1f, 5xdq.

## Supplementary Material

gkaa1032_Supplemental_Files
