# Supplementary material for: MitImpact 3: modeling the residue interaction network of the Respiratory Chain subunits
Source: Nucleic Acids Res. 2020 Dec 9;49(D1):D1282–8. doi: 10.1093/nar/gkaa1032 (PMC7779045; doi:10.1093/nar/gkaa1032)
Supplement: gkaa1032_Supplemental_Files [file gkaa1032_supplemental_files.zip › Supplementary_File_1.docx]

**Supplementary File 1A – Methods**

**Co-variation analysis**

In the last two decades, great efforts were made to assess the phenomenon of co-variation (concerted variation) occurring in biological sequences. This was made using classical (Mutual Information, e.g., (1)) and advanced (deep learning, e.g., (2)) analytical techniques.

As for many bioinformatics research areas, this topic bumped against three main issues:

- availability and quality of experimental molecular data (e.g., genomes, genomic variants, protein crystal structures);
- configuration, usability, and technical requirements (software, hardware) of the toolset;
- lack of extensive benchmarking studies, standard procedures, or standard file formats for input and output data.

In this work, we used tools that were (i) easy to use and configure; (ii) fast and efficient in generating results; (iii) capable of automatically generating concatenated alignments, where each sequence was then the concatenation of two aligned sequences of the same species; (iv) designed to output raw text output (*i.e.*, single protein alignments, concatenated alignments, raw co-variation score matrices).

We sought for these features in all available tools, such as (3-8), and we opted to use I-COMS for several reasons: (i) it generates concatenated alignments by only providing protein accession numbers; (ii) it can generate alignments taxonomically; (iii) it is equipped with four different algorithms for co-variation analysis; (iv) it is fast (results are produced in few hours) and output results in plain text.

Hence, we proceeded as follows:

- In the submission page of I-COMS (<http://i-coms.leloir.org.ar/>), we inserted UNIPROT (9) IDs for the pairs of the 13 Respiratory Chain mitochondrial subunits specified in the table below:

| Protein ID A (symbol) | Protein ID B (symbol) | Batch run | RC Complex |
| --- | --- | --- | --- |
| P03886 (MT-ND1) | P03891 (MT-ND2) | 1 | I, Inter-protein |
| P03886 (MT-ND1) | P03897 (MT-ND3) | 2 | I, Inter-protein |
| P03886 (MT-ND1) | P03905 (MT-ND4) | 3 | I, Inter-protein |
| P03886 (MT-ND1) | P03901 (MT-ND4L) | 4 | I, Inter-protein |
| P03886 (MT-ND1) | P03915 (MT-ND5) | 5 | I, Inter-protein |
| P03886 (MT-ND1) | P03923 (MT-ND6) | 6 | I, Inter-protein |
| P03891 (MT-ND2) | P03897 (MT-ND3) | 7 | I, Inter-protein |
| P03891 (MT-ND2) | P03905 (MT-ND4) | 8 | I, Inter-protein |
| P03891 (MT-ND2) | P03901 (MT-ND4L) | 9 | I, Inter-protein |
| P03891 (MT-ND2) | P03915 (MT-ND5) | 10 | I, Inter-protein |
| P03891 (MT-ND2) | P03923 (MT-ND6) | 11 | I, Inter-protein |
| P03897 (MT-ND3) | P03905 (MT-ND4) | 12 | I, Inter-protein |
| P03897 (MT-ND3) | P03901 (MT-ND4L) | 13 | I, Inter-protein |
| P03897 (MT-ND3) | P03915 (MT-ND5) | 14 | I, Inter-protein |
| P03897 (MT-ND3) | P03923 (MT-ND6) | 15 | I, Inter-protein |
| P03905 (MT-ND4) | P03901 (MT-ND4L) | 16 | I, Inter-protein |
| P03905 (MT-ND4) | P03915 (MT-ND5) | 17 | I, Inter-protein |
| P03905 (MT-ND4) | P03923 (MT-ND6) | 18 | I, Inter-protein |
| P03901 (MT-ND4L) | P03915 (MT-ND5) | 19 | I, Inter-protein |
| P03901 (MT-ND4L) | P03923 (MT-ND6) | 20 | I, Inter-protein |
| P03915 (MT-ND5) | P03923 (MT-ND6) | 21 | I, Inter-protein |
| P00395 (MT-CO1) | P00403 (MT-CO1) | 22 | IV, Inter-protein |
| P00395 (MT-CO1) | P00414 (MT-CO1) | 23 | IV, Inter-protein |
| P00403 (MT-CO2) | P00414 (MT-CO3) | 24 | IV, Inter-protein |
| P00846 (MT-ATP6) | P03928 (MT-ATP8) | 25 | V, Inter-protein |
| P03886 (MT-ND1) | P03886 (MT-ND1) | 26 | Intra-protein |
| P03891 (MT-ND2) | P03886 (MT-ND1) | 27 | Intra-protein |
| P03897 (MT-ND3) | P03886 (MT-ND1) | 28 | Intra-protein |
| P03905 (MT-ND4) | P03886 (MT-ND1) | 29 | Intra-protein |
| P03901 (MT-ND4L) | P03886 (MT-ND1) | 30 | Intra-protein |
| P03915 (MT-ND5) | P03886 (MT-ND1) | 31 | Intra-protein |
| P03923 (MT-ND6) | P03886 (MT-ND1) | 32 | Intra-protein |
| P00395 (MT-CO1) | P03886 (MT-ND1) | 33 | Intra-protein |
| P00403 (MT-CO2) | P03886 (MT-ND1) | 34 | Intra-protein |
| P00414 (MT-CO3) | P03886 (MT-ND1) | 35 | Intra-protein |
| P00846 (MT-ATP6) | P03886 (MT-ND1) | 36 | Intra-protein |
| P03928 (MT-ATP8) | P03886 (MT-ND1) | 37 | Intra-protein |
| P00156 (CYB) | P03886 (MT-ND1) | 38 | Intra-protein |

- We have then analyzed the entire amino acid sequences of both investigated proteins of the pairs, but restricting the search for co-varying sites to protein orthologs in the Mammalian clade (NCBI37 Taxon ID: 40674);
- We used two of the four available algorithms: cMI (10) and EVFold-mfDCA (11,12).

Twenty-one pairwise analyses were carried out for the Complex I, three for Complex IV (MT-CO1 + MT-CO2, MT-CO2 + MT-CO3, MT-CO1 + MT-CO3), one for Complex V (MT-ATP6 + MT-ATP8). These were named "inter-protein co-variation" analyses.

Moreover, thirteen "intra-protein co-variation" analyses were performed. It is important to note that we used the MT-ND1 protein as "Protein B" input for all analyses because I-COMS requires to input at least two distinct proteins to work correctly. The usage of MT-ND1 as a common partner in the intra-protein analyses is arbitrary. There are no strictly biological reasons for that. However, using the same protein as a partner of all other proteins certainly contributed to reducing the background noise that our analyses would have indeed suffered if using diverse partner proteins and discarding the same MT-ND1 site pairs from the pool of the top500 high scoring co-varying sites for all protein pairs.

Thus, we obtained the cMI and mfDCA matrix scores for each protein pair, which reported the co-variation scores of all pairs of sites within the paired proteins. We ranked the site pairs by their scores, selected the highest 500, and printed to files (*.gs.mi and *.gs.dca files for cMI and mfDCA analyses). The choice of selecting 500 site pairs, which is an I-COMS option, after all, was arbitrarily taken to have a reasonably large number of candidate pairs to be further investigated energetically (next step). From each pool of pairs, we dropped the pairs (s_A_, s_B_) when s_A_ was not located within the first *k* sites of the concatenated sequence of the protein A (*k* is the amino acid sequence length of the protein A), or s_B_ was not located within the last *j* sites of the concatenated sequence of the protein B (*j* is the amino acid sequence length of the protein B). This filtering step was performed using the "reference_sequence" and "label_map" comment lines contained in the "*.gs.mi" and "*.gs.dca" files. Regarding the intra-protein analyses, the top500 site pairs were filtered to retain only the pairs where s_A_ and s_B_ were located within the first *k* sites.

**Example**

For example, we focus on **icoms_1** folder of the archive available from <https://mitimpact.css-mendel.it/static/supporting_data/ICOMS_mitimpact3.rar>, and regarding the MT-ND1:MT-ND2 inter-protein analysis. I-COMS generated three alignments: "aln_01_01.fas.gs" for the MT-ND1 protein homologs (the first sequence, named "ORIGIN", is relative to the human MT-ND1 reference sequence, Uniprot accession: P03886); "aln_02_02.fas.gs" for the MT-ND2 protein homologs (the first sequence, named "ORIGIN", is relative to the human MT-ND2 reference sequence, Uniprot accession: P03891); "aln_01_02.fas.gs" for the reconstructed "MT-ND1:MT-ND2" protein alignment: the first sequence is named "ORIGIN|ORIGIN" (made by the concatenation of the MT-ND1 and MT-ND2 reference sequences); the second element, for example, is the concatenation of MT-ND1 and MT-ND2 from *Neofelis nebulosa* (leopard, NCBI Taxonomy ID: 61452). This particular alignment contains 878 *concatenated* MT-ND1:MT-ND2 protein sequences. I-COMS generated two co-variation matrices: "aln_01_02.fas.gs.mi", which is relative to the cMI method, and "aln_01_02.fas.gs.dca", which is relative to the mfDCA method.

A total of 220.780 possible site pairs were obtained for this pair, with a sequence length of, respectively, 318 and 347 amino acids. The total number of candidate site pairs was obtained with this formula:

$\frac{\left( k+j \right)^{2}-\left( k+j \right)}{2}$,

where *k* represents the amino acid length of the protein A, while *j* is the protein B amino acid length. Comment lines within files also report the minimum and maximum values for the scores plus the reference concatenated sequences. Scores are sorted in descending order, and only the 500 top-scored are reported. Each pair (s_A_, s_B_) guarantees that s_A_ is one of the first 317 sites, while s_B_ is located between sites 318 and 665 of the concatenated sequence. In **Supplementary Data 3**, the "cov inter" sheet, all the retained pairs are provided for all inter-protein analyses.

Notes on co-variation analysis

As in **Supplementary Data 3**, we compared the results obtained with the two I-COMS methods that we have used in this study. The "cov common" sheet lists the top-scored inter/intra-protein site pairs for all the considered protein pairs. Low overlap results from the two methods:

| Analysis | Total (cMI+mfDCA) | Common | Proportion of common pairs |
| --- | --- | --- | --- |
| Intra-protein | 6468 (3038+3430) | 339 | 5.2% |
| Inter-protein | 7017 (4285+2732) | 47 | 0.7% |

Low congruence would firstly depend on methodological differences. While Mutual Information is based on the co-occurrence of two events (*i.e.*, variations on site "A" and "B" of a sequence alignment) with respect to the frequency of every single event (however, several correction methods exist), mfDCA, which is an efficient version of Direct Coupling Analysis (13), relies on the idea that three-dimensional conformation imposes direct constraints on amino acid site variability.

Several works (14-16) have underlined the biological and methodological issues that influence the identification of co-varying sites within proteins. These are the phylogenetic bias (*i.e.*, the evolutionary dynamics of co-occurring variations can be unpredictable); sequence sampling (*i.e.*, specific clades are more sequenced and represented in biological databases); sequence and protein annotation quality; lack of resolved crystal structures; difficulty in distinguishing "direct" from "indirect" co-evolution (*i.e.*, site "1" is seen to co-evolve with "3", while true co-evolution are "1-2" and "2-3"); heterogeneity of statistical methods and score cutoffs to evidence "true" co-evolving sites from false positive.

To our knowledge, extensive and systematic comparative studies for this kind of algorithms have not been conducted yet. Tetchner et al. (15) evaluated the congruence of three popular methods (PSICOV, mfDCA, plmDCA). These tools' precision was around 70-80%, measured on a large series of Pfam protein domains, with resolved structures and extremely deep sequence coverage. Protein structure datasets ([https://predictioncenter.org/](https://predictioncenter.org/index.cgi)) have often been used to verify algorithm accuracy: the algorithm MetaPSICOV (17) reached an average precision of 27% on different protein benchmarks (the so-called "CASP11" protein sets). Deep MetaPSICOV (2) improved the prediction of contact sites in many "CASP13" datasets; however, many false positives, which implies low precision, were obtained for specific protein datasets.

In addition to methodological issues, poor congruence within our analysis could also be due to the choice of using a unique sorting and selection strategy for all site pairs. Given that we have incomplete knowledge of co-evolution in individual RC subunits and subunit pairs, we simply adopted general cutoff criteria to retrieve intra-protein or inter-protein site pairs for all protein comparisons. A better congruence would be achieved with a careful inspection of score distributions, trying to define a protein-specific cutoff score.

In this work, we were not interested to accurately find signals of amino-acid site co-evolution but to define two broad sets of candidate amino acid sites to be analyzed with FoldX downstream. In perspective, we will reconfigure our tools and calculate new measures of co-variation for all the above mentioned pairwise comparisons. We will plan to use the same multi-alignments and, secondarily, to produce new ones to examine the "sequence coverage" effect. We will try to define more accurate protein-specific thresholds to detect the right coevolving candidates from the thousands of site pair scores. Ultimately, we will use site-to-site co-variation measures as additional features for a new updated version of our missense variant classifier, APOGEE (18).

Data availability

Data were produced with I-COMS from 2017-2020. These were collected in folders named "icoms_N". Folders with N ranging from 1 to 25 contain inter-protein data. Each folder contains files whose names follow these patterns:

“aln_01_01.fas.gs” contains the alignment for protein 1

“aln_02_02.fas.gs” contains the alignment for protein 2

“aln_01_02.fas.gs” contains the concatenated alignment for protein 1 and 2

“aln_01_02.fas.gs.dca” contains the raw scores obtained with the mfDCA method

“aln_01_02.fas.gs.mi” contains the raw scores obtained with the cMI method.

Folders from 26 to 38 contain intra-protein data. An archive containing all these folders is available from <https://mitimpact.css-mendel.it/static/supporting_data/ICOMS_mitimpact3.rar>

**Energetic Analysis by FoldX**

For each mtDNA-encoded protein site pairs obtained in the previous step, the overall stability energy and binding affinity changes upon mutations were calculated from a structural standpoint. In fact, amino acid substitutions located at the protein-protein interface are tagged as deleterious when they significantly impair the binding affinity. However, these may also exhibit potentially compensatory mechanisms when occurring in a group that could attenuate or abolish individual variants' harmful effects. A full understanding of how these mechanisms occur requires optimal three-dimensional proteic structures, as they provide crucial atomic details about binding, and then describing protein-protein interfaces.

SwissModel (19) was employed to predict the best possible three‐dimensional model starting from the human mtDNA-encoded RC proteins sequences and then using homology template structures available from the Protein Data Bank. The obtained models but MT-ATP8, which was excluded from any further analysis, showed a sequence identity > than 70% and a sequence similarity > 50%. Moreover, coverage values were above 90%, indicating that most human structures were successfully recreated.

Known RC interacting protein-protein pairs were retrieved by the related *Bos taurus* structures since this was the evolutionary closest Mammalia whose proteins were stored in the Protein Data Bank at the time of this analysis (PDB IDs: *1be3, 1bgy, 1bmf, 1cow, 1e1q, 1e1r, 1e79, 1efr, 1h8e, 1h8h, 1l0l, 1l0n, 1mab, 1nbm, 1ntk, 1ntm, 1ntz, 1nu1, 1occ, 1oco, 1ocr, 1ocz, 1ohh, 1pp9, 1ppj, 1qcr, 1qo1, 1sqb, 1sqp, 1sqq, 1sqv, 1sqx, 1v54, 1v55, 1w0j, 1w0k, 2a06, 2bcc, 2ck3, 2cly, 2dyr, 2dys, 2eij, 2eik, 2eil, 2eim, 2ein, 2f43, 2fyu, 2jdi, 2jiz, 2jj1, 2jj2, 2occ,2v7q, 2w6e, 2w6f, 2w6g, 2w6h, 2w6i, 2w6j, 2wss, 2xnd, 2y69, 2ybb, 2zxw, 3abk, 3abl, 3abm, 3ag1, 3ag2, 3ag3, 3ag4, 3asn, 3aso, 3bcc, 3wg7, 3x2q, 4asu, 4b2q, 4d6t, 4d6u, 4tsf, 4tt3, 4yxw, 4z1m, 5ara, 5are, 5arh, 5ari, 5b1a, 5b1b, 5b3s, 5fij, 5fik, 5fil, 5gpn, 5iy5, 5klv, 5lc5, 5ldw, 5ldx, 5lnk, 5luf,5nmi, 5w97, 5wau, 5x19, 5x1b, 5x1f, 5xdq*). The MatchMaker extension of UCSF Chimera (20) was used to recreate by superimposition the interactions between proteins.

Then, we performed a minimization step conducted into the membrane environment to refine the interface regions and reduce local errors, such as clashes and Ramachandran outliers. In brief, each protein-protein complex was embedded into a POPC:POPE 3:1 lipid bilayer using the PackMol-MemGen tool (21), inserted in a box filled with TIP3P water molecules, and then subjected to 5.000 steps of energy minimization using the steepest descent method, followed by the *conjugate-gradient* method and using the *sander* program of AMBER 18 (22).

The refined models and all intra-protein and inter-protein co-varying sites located at the protein-protein interface were given in input to FoldX. According to the FoldX suggested workflow, ten preliminary steps of RepairPDB were applied to achieve full relaxation of both individual and protein-protein complex models. Moreover, the *–membrane* flag was set to *True* for the sites that were predicted using the TMHMM tool (23) to form a transmembrane helix in our proteins to switch off the hydrophobic contributions and set the dielectric constant to zero. In detail, this parameter allows us to reproduce a membrane environment by appropriately modifying the dielectric constant in order to evaluate the energy contributions of the molecules located into the inner mitochondrial membrane. Co-varying sites located one in the transmembrane region and one exposed in the outer surface were not included in this energetic analytical step since FoldX allows to set a unique dielectric constant for the whole system.

Finally, BuildModel was employed to mutate the residues given in input into all the possible amino acid substitutions. AnalyseComplex was used only with the inter-proteins sites to determine the ΔΔG of interaction; whereas for the intra-protein sites, only the ΔΔG of stability was evaluated. Amino acid changes causing a ΔΔG to exceed the suggested cutoff (±0.61 Kcal/mol) were tagged as candidate disrupting mutations. Then, pairs of mutants with a joint ΔΔG close to 0 (< ±0.1 Kcal/mol) has been considered as co-interacting and potentially compensative.

**Molecular Dynamics Simulations**

The free-energy calculation represents a rapid and efficient method to evaluate mutations' effect on protein stability or protein-protein interactions. However, proteins are flexible molecules that undergo conformational fluctuations and changes that require advanced and more complex analytical techniques to be captured and assessed. The systems modeling proteins are characterized by complex interaction mechanisms that cannot be immediately ascribed to individual components. However, they are often derived from group effects, *i.e.*, systemic behaviors do not result from an individual element's action, but from the harmonized effects of interacting elements.

Molecular dynamics (MD) simulation represents an advanced computational approach to predict how atoms move over time based on a general physical model that governs the interatomic interactions. An important application of MD simulation is that of determining how a biomolecular system responds to perturbations. Amino acid variants can be considered harmful perturbations when they significantly destabilize the protein or its interactions with other proteins, making the protein complex partly or entirely non-functional, or worse, aberrantly functioning. However, compensatory mechanisms may occur that restore the whole protein conformation to that of the native protein, thereby preserving the protein's biological functions.

Based on these considerations, we selected the putatively co-varying pairs of residues of which at least one of the pair was reported as pathogenic in MITOMAP, and both were located in the interaction interface of proteins belonging to the same RC complex. Then, we mapped these residues onto the recently resolved Cryo-EM structure of the human respiratory complex I transmembrane arm (PDB code: 5xtc (24)) and investigated the interacting properties of the wild-type protein complex as well as of the single and double-mutated complexes through 50 ns-long classical molecular dynamics simulations performed using AMBER 18.

System preparation and MD setup

Atomic coordinates of interacting RC proteins MT-ND1:MT-ND3 (chain *s* and *j*, respectively) and MT-ND4L/MT-ND6 (chain *m* and *k*) were isolated from the 5xtc complex. Then, these wild-type structures were mutated in-silico to introduce all the possible combinations of amino acids changes for each selected co-varying pairs, both single and double mutants.

The obtained protein complexes were embedded into a POPC:POPE 3:1 lipid bilayer generated using the PackMol-MemGen tool. The titratable groups of the protein were protonated according to their standard protonation states at pH 7. The system was then solvated using the TIP3P water model, Na+ and Cl- ions at physiological saltcon of 0.15 M. Each simulation box was subjected to 5.000 steps of energy minimization using the steepest descent method, followed by the *conjugate-gradient* method. The system was then gradually heated and then equilibrated for approximately 5 ns, by time steps of 1 fs. A 10 Å cutoff was used for non-bonded short-range interactions, while long-range electrostatics were treated with the particle-mesh Ewald method. Temperature and pressure were maintained at 300 K and 101.3 kPa, respectively, using the Langevin dynamics and Piston method. The last frame of equilibration was extracted and subjected to classical molecular dynamics simulation. Ten replicas of simulation of the wild-type, single and double mutant proteins were performed for each pair. All computations were performed on a GPU cluster equipped with three NVIDIA RTX 2080ti and one NVIDIA^©^ Quadro P6000.

MD simulation analysis

The root-mean-square deviation (RMSD) and the root mean square fluctuation (RMSF), measuring respectively the average distance and the deviation over time between the positions of the Cα atomic coordinates of each residue and those of the reference X-ray structure, were measured on the simulated trajectories using GROMACS 4.5 (25). Moreover, the GROMACS plugin g_hbond was used to evaluate the per-residue hydrogen bonds, with an angle cutoff of 30° and a donor-acceptor distance of 3.5 Å. Finally, the essential protein motions' collective coordinates were inferred by principal component analysis of the atomic fluctuations. The 3D movies of the molecular dynamics simulations were obtained using the *g_anaeig* GROMACS tool.

To estimate the binding energy during the simulations, we used the *Molecular mechanics Poisson–Boltzmann surface area* (MM-PBSA) approach implemented in the *g_mmpbsa* package (26).

**Supplementary File 1B – CPD Pipeline description**

The Compensated Pathogenic Deviations (CPD) are deleterious human mutations found as reference alleles in non-human homolog proteins. Systematic computational methods (27-29) applied either on nuclear or mitochondrial non-synonymous variants predicted that 3-10% of the harmful amino acid changes are present as wild-type residues into other eukaryotic species. CPD mutations showed to be fixed in closely-related species, in particular, which exhibited the necessary "compensatory" genetic backgrounds in order to mask or suppress their pathological effects. The identification of CPDs is mainly carried out through alignment-based bioinformatics pipelines, while investigating variant pathogenicity and compensation in non-human context is technically difficult, beyond being costly and time expensive. Our pipeline is depicted in **Figure 1**.


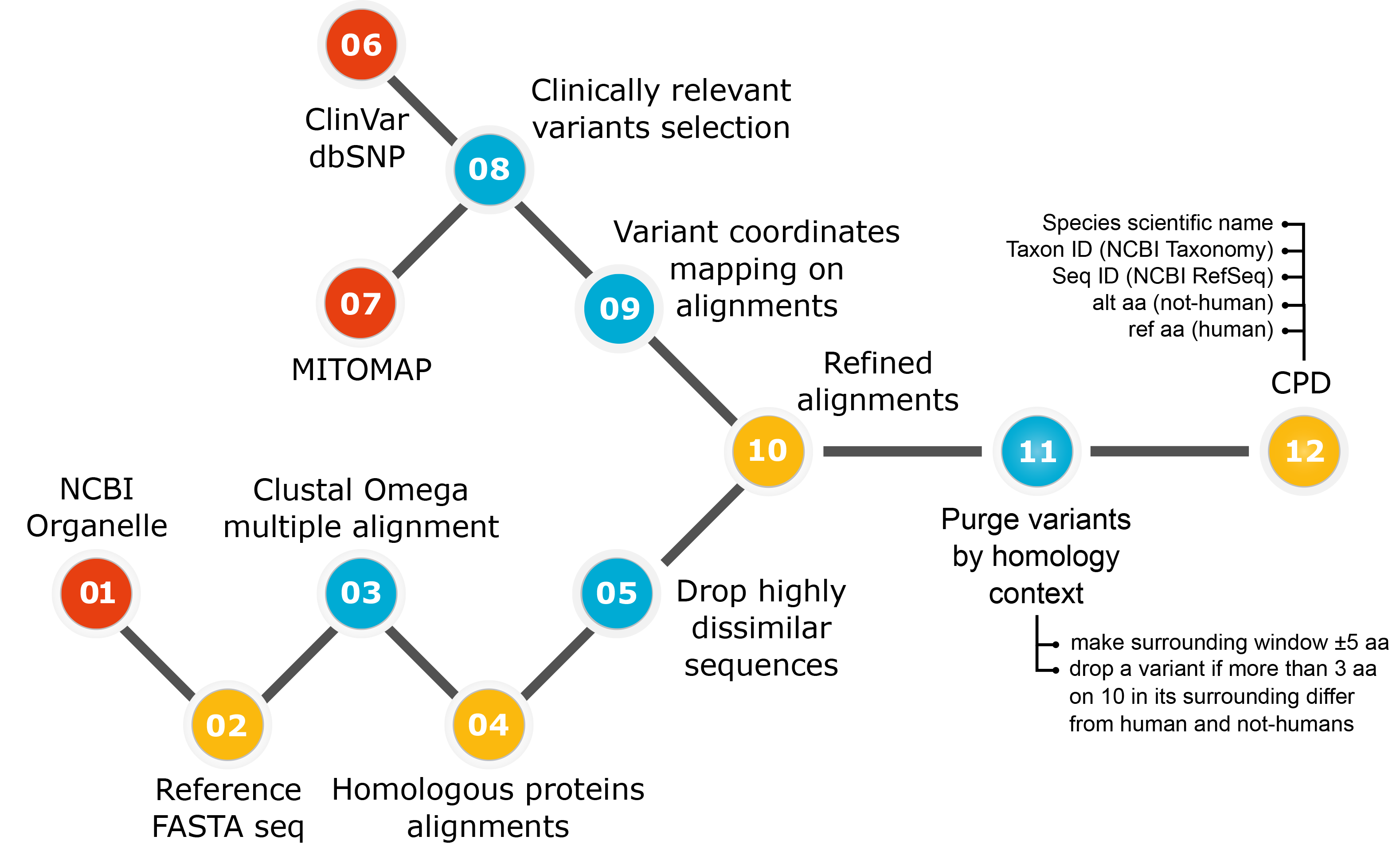


**Figure 1**: Representation of the implemented analysis pipeline to find putatively CPDs. Red circles represent data sources or databases; orange circles represent data retrieved from data sources or databases; blue circles represent actions performed.

We initially downloaded the reference sequences for the 13 mitochondrial RC subunits from the NCBI Organelle resource (<https://www.ncbi.nlm.nih.gov/genome/organelle/>, accessed in Spring 2017), considering only the *Mammalia* taxon. Thus, we aligned the homologous sequence sets with ClustalOmega (<https://www.ebi.ac.uk/Tools/msa/clustalo/>, accessed in Spring 2017), obtaining a set of "Homologous protein alignments". An archive of the resulting alignments is available from <https://mitimpact.css-mendel.it/static/supporting_data/CPD_ClustalOmega_mitimpact3.rar>.

Then, we proceeded to drop highly dissimilar sequences by removing sequences with <50% similarity with our reference system, the human mitochondrial proteins (27). 30% similarity was set as a threshold for the MT-ATP8 protein since purifying selection is milder, then inter-species variability is higher for this protein (30).

Thus, we collected and revised a list of 255 clinically relevant non-synonymous variants from dbSNP-ClinVar and MITOMAP databanks, accessed in Spring 2017. We used the R *seqinr* (31) package to scan the 13 mitochondrial RC subunits' alignments and determine the relative positions of all these variants. We then refined the alignments, filtering only those carrying the human alternate deleterious amino acids as reference alleles. We considered a window made by the five neighbor amino acids upstream and downstream for each of these variants and discarded those whose windows were not similar between non-human and human sequences for at least seven amino acids (27). The surviving 41 variants were reported in **Supplementary Data 2**.

A precise analytical strategy for defining and quantifying CPDs along genomes is still a matter of debate. The taxonomic context should be large enough to contain sufficient genomic variability to explore, but should not include species too much phylogenetically distant from humans. Highly diverging proteins could be dramatically different in structure and function; thus, an amino acid residue's impact in a given species could not be comparable with the same residue's effect in humans. The extension of the variant flanking regions, sequence similarity cutoffs, and orthology/paralogy issues are debated because protein families have specific evolutionary trajectories. Some uncertainty also regards the definition of the human pathogenic set to screen since it evolves over time, new disease associations are found or confuted, and disease categories (*i.e.*, mendelian monogenic, mitochondrial, cancer, multi-factorial diseases) are debated to be included.

**Supplementary File 1C – Case study**

Leigh Disease is a very rare and severe mitochondrial disorder that arises in the first months of life; it has been associated with at least 75 nuclear and mitochondrial-encoded genes with functional implications in mitochondrial biogenesis and metabolism (32). The disease is characterized by neurological regression with specific clinical lesions at basal ganglia and brainstem levels.

A mutation in the ND3 gene, **m.10191T>C**, has been associated with severe Complex I deficiency and neurological degeneration in adult patients (33) and children with Leigh disease (34,35). The mutation encodes for a Serine-Proline substitution in position 45. This missense variant is classified as "Confirmed" pathogenic mutation in MITOMAP, with Leigh Disease and Leigh Disease-like associated phenotypes.

**We asked ourselves if theoretical compensatory variants exist for such mutation, within the same protein or in the interaction interface with another mitochondrial protein.**

As a first step, we obtained 87 possibly inter-protein co-evolving pairs involving the site 45 of ND3. We considered only the pairs mapping on the physical interface between ND3 and ND1 and retrieved three PDB structures that resolved their interaction interface. Then, we calculated the ∆∆G of binding energy through the FoldX suite. ∆∆G_A_ is the binding energy variation of the S45P mutant concerning the wild type ND3 protein; ∆∆G_B_ is the partner mutant's binding energy variation for the wild type ND1 protein; ∆∆G_AB_ is the binding energy variation when both proteins are mutated at their interaction interface. Note that ∆∆G_A_ is below the conventional threshold of ±0.61 Kcal/mol that identifies the "disruptive" variants for two PDB complexes, **5lc5** and **5ldx**. We evidenced that three amino acid changes in the ND1 position 126 can restore the binding energy to, approximately, wild type levels (row in bold characters in the table below). Indeed, ∆∆G _AB_ is proximal to 0, indicating that the interface with two co-occurring variations has similar binding energy to the wild-type protein complex.

| **Interactor** | **PDB ID** | **Chain**  **(A protein)** | **Chain**  **(B protein)** | **Target variant**  **(A)** | **Partner variant (B)** | **∆∆G _AB_** | **∆∆G _A_** | **∆∆G _B_** |
| --- | --- | --- | --- | --- | --- | --- | --- | --- |
| MT-ND1 | 5lc5 | A | H | S45P | A64P | 2.340 | -1.091 | 3.391 |
| MT-ND1 | 5lc5 | A | H | S45P | A64D | -1.071 | -1.091 | 0.005 |
| MT-ND1 | 5lc5 | A | H | S45P | A64G | -1.410 | -1.091 | -0.339 |
| MT-ND1 | 5lc5 | A | H | S45P | A64V | -0.870 | -1.091 | 0.204 |
| MT-ND1 | 5lc5 | A | H | S45P | A64S | -1.098 | -1.091 | -0.015 |
| MT-ND1 | 5lc5 | A | H | S45P | A64T | -0.923 | -1.091 | 0.207 |
| MT-ND1 | 5lc5 | A | H | S45P | K62Q | -1.358 | -1.091 | -0.241 |
| MT-ND1 | 5lc5 | A | H | S45P | K62T | -0.341 | -1.091 | 0.832 |
| MT-ND1 | 5lc5 | A | H | S45P | K62M | -1.309 | -1.091 | -0.217 |
| MT-ND1 | 5lc5 | A | H | S45P | K62E | -0.630 | -1.091 | 0.412 |
| MT-ND1 | 5lc5 | A | H | S45P | K62N | -0.312 | -1.091 | 0.846 |
| **MT-ND1** | **5lc5** | **A** | **H** | **S45P** | **N126K** | **-0.063** | **-1.091** | **0.553** |
| MT-ND1 | 5lc5 | A | H | S45P | N126D | 1.607 | -1.091 | 1.595 |
| MT-ND1 | 5lc5 | A | H | S45P | N126I | -1.125 | -1.091 | 0.053 |
| **MT-ND1** | **5lc5** | **A** | **H** | **S45P** | **N126Y** | **0.031** | **-1.091** | **0.641** |
| MT-ND1 | 5lc5 | A | H | S45P | N126T | 0.129 | -1.091 | 0.124 |
| **MT-ND1** | **5lc5** | **A** | **H** | **S45P** | **N126S** | **0.097** | **-1.091** | **0.245** |
| MT-ND1 | 5lc5 | A | H | S45P | N126H | 0.487 | -1.091 | 0.694 |
| MT-ND1 | 5ldw | A | H | S45P | A64P | 1.517 | -0.288 | 1.892 |
| MT-ND1 | 5ldw | A | H | S45P | A64D | -0.286 | -0.288 | 0.016 |
| MT-ND1 | 5ldw | A | H | S45P | A64G | -0.586 | -0.288 | -0.290 |
| MT-ND1 | 5ldw | A | H | S45P | A64V | -0.201 | -0.288 | 0.074 |
| MT-ND1 | 5ldw | A | H | S45P | A64S | -0.314 | -0.288 | -0.001 |
| MT-ND1 | 5ldw | A | H | S45P | A64T | -0.184 | -0.288 | 0.101 |
| MT-ND1 | 5ldw | A | H | S45P | K62Q | -0.302 | -0.288 | -0.029 |
| MT-ND1 | 5ldw | A | H | S45P | K62T | -0.157 | -0.288 | 0.183 |
| MT-ND1 | 5ldw | A | H | S45P | K62M | -0.296 | -0.288 | -0.012 |
| MT-ND1 | 5ldw | A | H | S45P | K62E | 0.035 | -0.288 | 0.301 |
| MT-ND1 | 5ldw | A | H | S45P | K62N | 0.175 | -0.288 | 0.465 |
| MT-ND1 | 5ldw | A | H | S45P | N126K | -0.131 | -0.288 | -0.182 |
| MT-ND1 | 5ldw | A | H | S45P | N126D | 0.960 | -0.288 | 0.906 |
| MT-ND1 | 5ldw | A | H | S45P | N126I | -0.791 | -0.288 | 0.457 |
| MT-ND1 | 5ldw | A | H | S45P | N126Y | -0.186 | -0.288 | 0.152 |
| MT-ND1 | 5ldw | A | H | S45P | N126T | -0.684 | -0.288 | -0.612 |
| MT-ND1 | 5ldw | A | H | S45P | N126S | -0.479 | -0.288 | -0.494 |
| MT-ND1 | 5ldw | A | H | S45P | N126H | 0.112 | -0.288 | 0.233 |
| MT-ND1 | 5ldx | A | H | S45P | A64P | 4.036 | 0.854 | 3.812 |
| MT-ND1 | 5ldx | A | H | S45P | A64D | 0.685 | 0.854 | -0.011 |
| MT-ND1 | 5ldx | A | H | S45P | A64G | 0.442 | 0.854 | -0.325 |
| MT-ND1 | 5ldx | A | H | S45P | A64V | 0.603 | 0.854 | -0.005 |
| MT-ND1 | 5ldx | A | H | S45P | A64S | 0.748 | 0.854 | -0.004 |
| MT-ND1 | 5ldx | A | H | S45P | A64T | 0.884 | 0.854 | 0.107 |
| MT-ND1 | 5ldx | A | H | S45P | K62Q | 0.700 | 0.854 | 0.015 |
| MT-ND1 | 5ldx | A | H | S45P | K62T | 0.634 | 0.854 | 0.109 |
| MT-ND1 | 5ldx | A | H | S45P | K62M | 0.680 | 0.854 | 0.050 |
| MT-ND1 | 5ldx | A | H | S45P | K62E | 1.136 | 0.854 | 0.575 |
| MT-ND1 | 5ldx | A | H | S45P | K62N | 1.255 | 0.854 | 0.452 |
| MT-ND1 | 5ldx | A | H | S45P | N126K | 0.519 | 0.854 | 0.152 |
| MT-ND1 | 5ldx | A | H | S45P | N126D | 1.809 | 0.854 | 1.314 |
| MT-ND1 | 5ldx | A | H | S45P | N126I | 0.203 | 0.854 | 0.753 |
| MT-ND1 | 5ldx | A | H | S45P | N126Y | 0.866 | 0.854 | 0.512 |
| MT-ND1 | 5ldx | A | H | S45P | N126T | 0.133 | 0.854 | -0.200 |
| MT-ND1 | 5ldx | A | H | S45P | N126S | 0.433 | 0.854 | -0.153 |
| MT-ND1 | 5ldx | A | H | S45P | N126H | 1.079 | 0.854 | 0.557 |

The effect of ND3-ND1 interactions can also be observed in the Molecular Dynamics section of the MitImpact website (<https://mitimpact.css-mendel.it/dynamics>). **MD16**, **MD29,** and **MD31** collect results obtained by MD simulations of the amino acid pairs involving the positions 45 and 126 in ND3 and ND1, respectively.

Some interesting results emerge when studying the S45P-N126S variant pairs. The RMSD plot shows how the S45P mutant subunit remains stably compact over the simulation time (i.e., lower RMSD) with respect to the wild-type protein complex (in red). The RMSD of the double mutant neatly overlaps that of the wild-type complex. Focusing on the RMSF plot, we observe high residue fluctuations from the positions 20 to 50 in the ND3 protein (chain B). The S45P mutation causes lower mobility of that region, while the occurrence of both mutations on the proteins interface (blue line) partially restores the wild-type condition (red line).

Detailed information on the S45P variant can be visualized on the MitImpact web site. Below, a brief hands-on tutorial is given.

1. Query the webpage <http://mitimpact.css-mendel.it>, selecting "Search by Gene or Protein position" and clicking on the "Gene symbol" button.
2. Then, select "MT-ND3" in the drop-down menu, type "45" as "Position" and click on the "Submit" button.


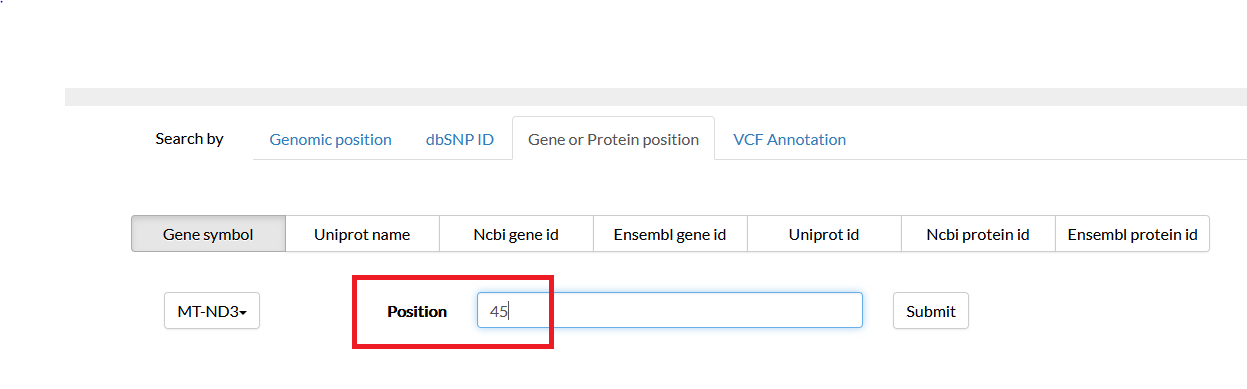


1. The results page will show all possible genomic variants encoding the ND2 amino acid changes at 45 position.
2. Choose the genomic variant of interest. In this case, click on "10191 (T>C)".


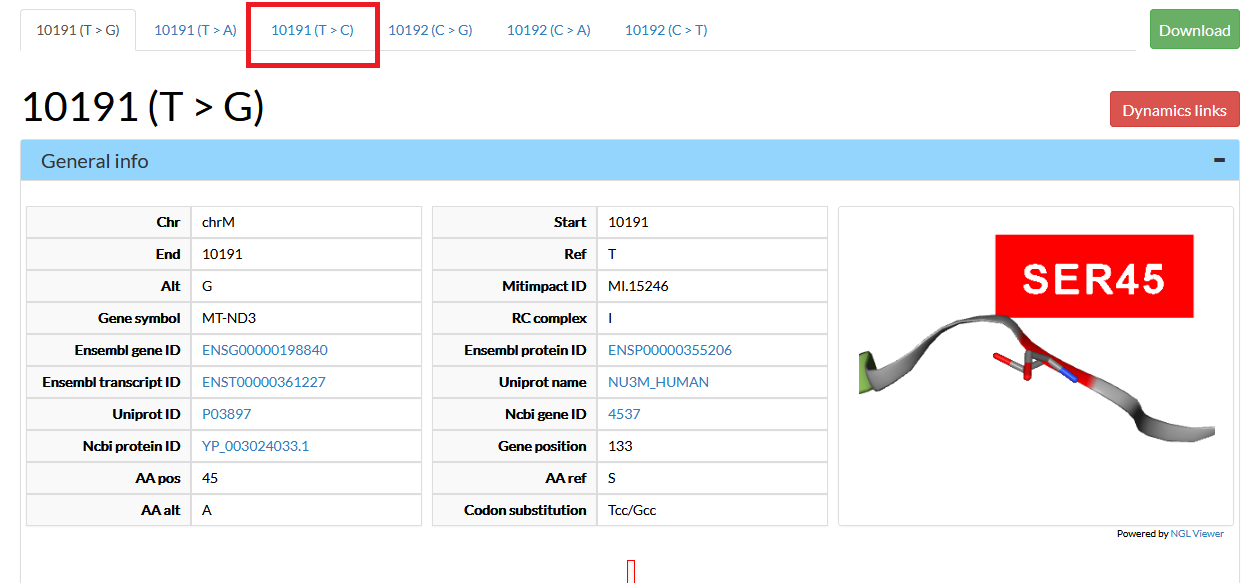


1. The results page will be updated with data concerning the mutation **10191T>C** and encoding for the S45P amino acid change.
2. Looking at the "Pathogenicity predictors" section, one can visualize pre-computed pathogenicity assessments from many computational algorithms. Clicking on the “?” symbol, one obtains details about scores and their interpretations. Note that score thresholds are not “dogmatic”, i.e., they do not absolutely separate neutral from pathogenic variants. Given the heterogeneity of predictions, we suggest considering some of the recent meta-predictors (APOGEE, Deogen2, MtoolBox, Meta-SNP) to classify variants' pathogenicity.


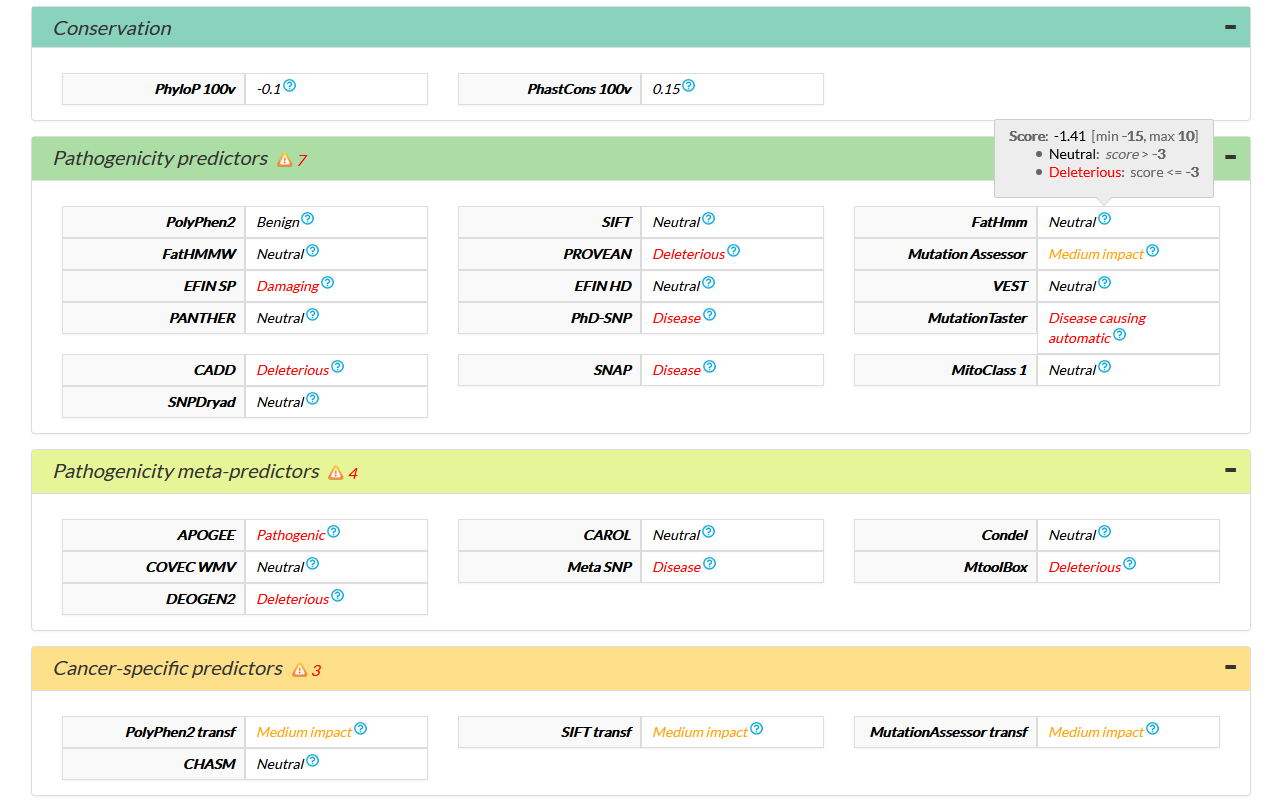


1. MitImpact 3 deals also with binary interactions between amino acids in the new "Residue interaction" section. There, you can visualize the ∆∆G for single and wild-type protein mutants through the drop-down menus labeled **∆∆G intra**, **∆∆G intra interface**, and **∆∆G inter**. The first variant pair in the ∆∆G inter menu is ND3 S45P *vs.* ND1 A64P (10), which was studied in the **5lc5** PDB structure. ∆∆G 1st and ∆∆G 2nd are both > |0.61|; then, they significantly alter the binding energy of the complex. ∆∆G both for the double mutant is 2.34 Kcal/mol, very far from 0, meaning that the native ∆∆G is not restored by the co-occurrence of the two mutations (S45P and A64P).


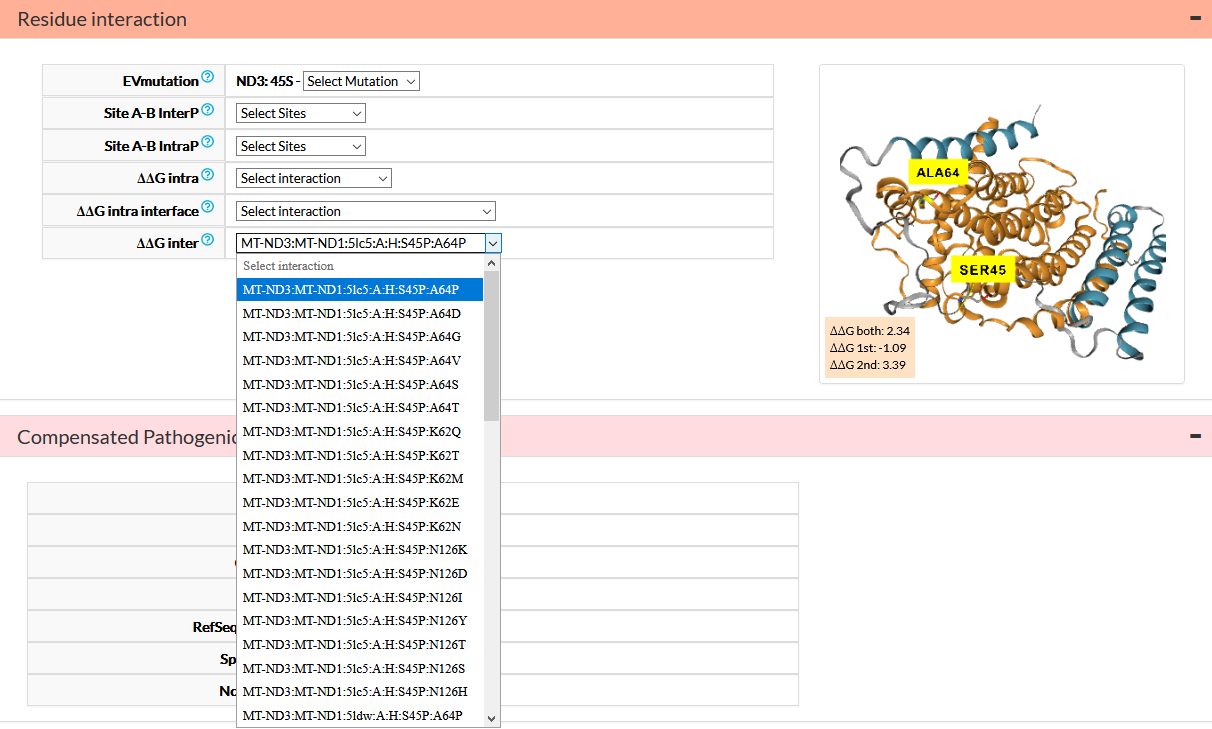


1. Selecting the partner N126S, instead, we can see that the S45P variant (∆∆G 1st) considerably alters the binding energy (-1.09 Kcal/mol)(13), while N126S on the ND1 subunit has a lower effect (∆∆G 2nd = 0.25)(13). The global effect of the two mutations in the ND3-ND1 interface is ∆∆G both = 0.1(13). We recall that if -0.1 ≤ ∆∆Gboth ≤ 0.1, then the two variants can be considered structurally compensating.


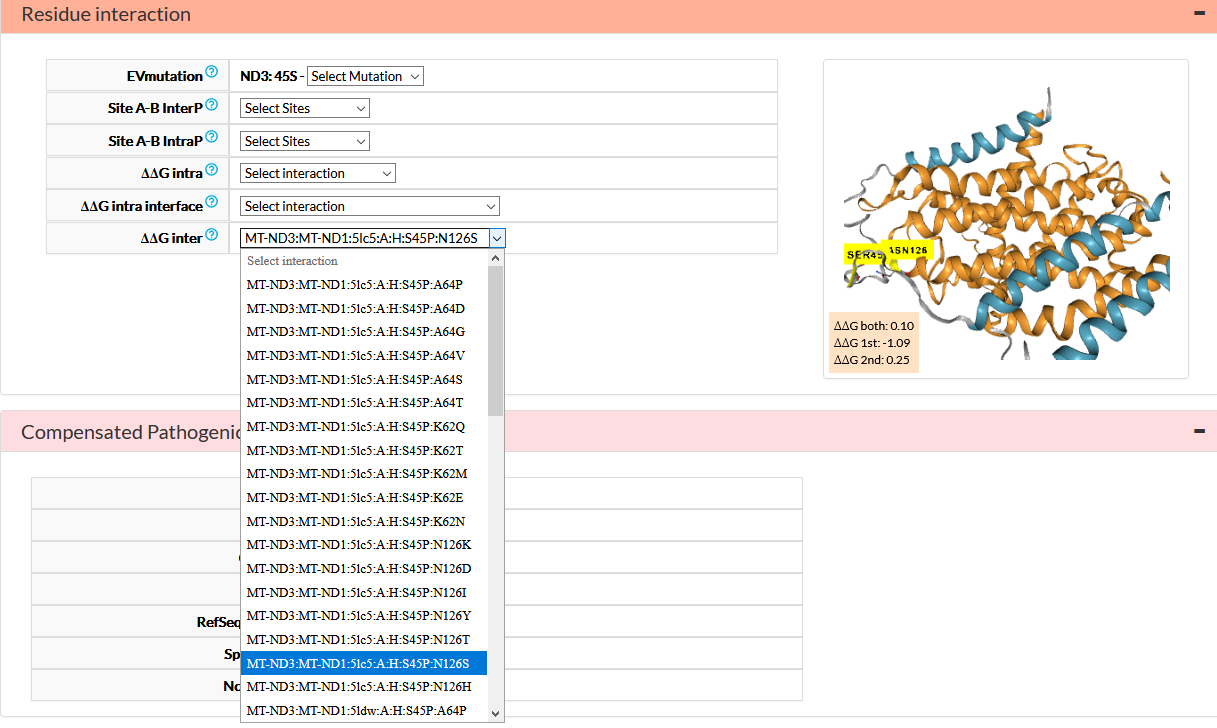


The potential compensatory effects on interesting missense variants can thus be retrieved by exploring the “Residue interaction” section. The users can download the flat file version of out MitImpact 3 database and evaluate the fields ΔΔG intraP, ΔΔG intraP interface, and ΔΔG interP, manually.

Further details can be found in the “Output legend” page (<https://mitimpact.css-mendel.it/result_legend>).

**Bibliography**

1. Martin, L.C., Gloor, G.B., Dunn, S.D. and Wahl, L.M. (2005) Using information theory to search for co-evolving residues in proteins. *Bioinformatics*, **21**, 4116-4124.

2. Kandathil, S.M., Greener, J.G. and Jones, D.T. (2019) Prediction of interresidue contacts with DeepMetaPSICOV in CASP13. *Proteins*, **87**, 1092-1099.

3. Simonetti, F.L., Teppa, E., Chernomoretz, A., Nielsen, M. and Marino Buslje, C. (2013) MISTIC: Mutual information server to infer coevolution. *Nucleic acids research*, **41**, W8-14.

4. Kozma, D., Simon, I. and Tusnady, G.E. (2012) CMWeb: an interactive on-line tool for analysing residue-residue contacts and contact prediction methods. *Nucleic acids research*, **40**, W329-333.

5. Meyer, X., Dib, L. and Salamin, N. (2019) CoevDB: a database of intramolecular coevolution among protein-coding genes of the bony vertebrates. *Nucleic acids research*, **47**, D50-D54.

6. Iserte, J., Simonetti, F.L., Zea, D.J., Teppa, E. and Marino-Buslje, C. (2015) I-COMS: Interprotein-COrrelated Mutations Server. *Nucleic acids research*, **43**, W320-325.

7. Yip, K.Y., Patel, P., Kim, P.M., Engelman, D.M., McDermott, D. and Gerstein, M. (2008) An integrated system for studying residue coevolution in proteins. *Bioinformatics*, **24**, 290-292.

8. Oteri, F., Nadalin, F., Champeimont, R. and Carbone, A. (2017) BIS2Analyzer: a server for co-evolution analysis of conserved protein families. *Nucleic acids research*, **45**, W307-W314.

9. The UniProt, C. (2017) UniProt: the universal protein knowledgebase. *Nucleic acids research*, **45**, D158-D169.

10. Buslje, C.M., Santos, J., Delfino, J.M. and Nielsen, M. (2009) Correction for phylogeny, small number of observations and data redundancy improves the identification of coevolving amino acid pairs using mutual information. *Bioinformatics*, **25**, 1125-1131.

11. Kajan, L., Hopf, T.A., Kalas, M., Marks, D.S. and Rost, B. (2014) FreeContact: fast and free software for protein contact prediction from residue co-evolution. *BMC bioinformatics*, **15**, 85.

12. Marks, D.S., Colwell, L.J., Sheridan, R., Hopf, T.A., Pagnani, A., Zecchina, R. and Sander, C. (2011) Protein 3D structure computed from evolutionary sequence variation. *PloS one*, **6**, e28766.

13. Morcos, F., Pagnani, A., Lunt, B., Bertolino, A., Marks, D.S., Sander, C., Zecchina, R., Onuchic, J.N., Hwa, T. and Weigt, M. (2011) Direct-coupling analysis of residue coevolution captures native contacts across many protein families. *Proceedings of the National Academy of Sciences of the United States of America*, **108**, E1293-1301.

14. Dickson, R.J. and Gloor, G.B. (2014) Bioinformatics identification of coevolving residues. *Methods in molecular biology*, **1123**, 223-243.

15. Tetchner, S., Kosciolek, T. and Jones, D.T. (2014) Opportunities and limitations in applying coevolution-derived contacts to protein structure prediction. *Bio-Algorithms and Med-Systems*, **10**, 243–254.

16. Nicoludis, J.M. and Gaudet, R. (2018) Applications of sequence coevolution in membrane protein biochemistry. *Biochimica et biophysica acta. Biomembranes*, **1860**, 895-908.

17. Kosciolek, T. and Jones, D.T. (2016) Accurate contact predictions using covariation techniques and machine learning. *Proteins*, **84 Suppl 1**, 145-151.

18. Castellana, S., Fusilli, C., Mazzoccoli, G., Biagini, T., Capocefalo, D., Carella, M., Vescovi, A.L. and Mazza, T. (2017) High-confidence assessment of functional impact of human mitochondrial non-synonymous genome variations by APOGEE. *PLoS computational biology*, **13**, e1005628.

19. Biasini, M., Bienert, S., Waterhouse, A., Arnold, K., Studer, G., Schmidt, T., Kiefer, F., Gallo Cassarino, T., Bertoni, M., Bordoli, L. *et al.* (2014) SWISS-MODEL: modelling protein tertiary and quaternary structure using evolutionary information. *Nucleic acids research*, **42**, W252-258.

20. Pettersen, E.F., Goddard, T.D., Huang, C.C., Couch, G.S., Greenblatt, D.M., Meng, E.C. and Ferrin, T.E. (2004) UCSF Chimera--a visualization system for exploratory research and analysis. *Journal of computational chemistry*, **25**, 1605-1612.

21. Schott-Verdugo, S. and Gohlke, H. (2019) PACKMOL-Memgen: A Simple-To-Use, Generalized Workflow for Membrane-Protein-Lipid-Bilayer System Building. *Journal of chemical information and modeling*, **59**, 2522-2528.

22. Case, D.A., Cheatham, T.E., 3rd, Darden, T., Gohlke, H., Luo, R., Merz, K.M., Jr., Onufriev, A., Simmerling, C., Wang, B. and Woods, R.J. (2005) The Amber biomolecular simulation programs. *Journal of computational chemistry*, **26**, 1668-1688.

23. Krogh, A., Larsson, B., von Heijne, G. and Sonnhammer, E.L. (2001) Predicting transmembrane protein topology with a hidden Markov model: application to complete genomes. *Journal of molecular biology*, **305**, 567-580.

24. Guo, R., Zong, S., Wu, M., Gu, J. and Yang, M. (2017) Architecture of Human Mitochondrial Respiratory Megacomplex I2III2IV2. *Cell*, **170**, 1247-1257 e1212.

25. Pronk, S., Pall, S., Schulz, R., Larsson, P., Bjelkmar, P., Apostolov, R., Shirts, M.R., Smith, J.C., Kasson, P.M., van der Spoel, D. *et al.* (2013) GROMACS 4.5: a high-throughput and highly parallel open source molecular simulation toolkit. *Bioinformatics*, **29**, 845-854.

26. Kumari, R., Kumar, R., Open Source Drug Discovery, C. and Lynn, A. (2014) g_mmpbsa--a GROMACS tool for high-throughput MM-PBSA calculations. *Journal of chemical information and modeling*, **54**, 1951-1962.

27. Azevedo, L., Mort, M., Costa, A.C., Silva, R.M., Quelhas, D., Amorim, A. and Cooper, D.N. (2016) Improving the in silico assessment of pathogenicity for compensated variants. *European journal of human genetics : EJHG*, **25**, 2-7.

28. Jordan, D.M., Frangakis, S.G., Golzio, C., Cassa, C.A., Kurtzberg, J., Task Force for Neonatal, G., Davis, E.E., Sunyaev, S.R. and Katsanis, N. (2015) Identification of cis-suppression of human disease mutations by comparative genomics. *Nature*, **524**, 225-229.

29. Klink, G.V., Golovin, A.V. and Bazykin, G.A. (2017) Substitutions into amino acids that are pathogenic in human mitochondrial proteins are more frequent in lineages closely related to human than in distant lineages. *PeerJ*, **5**, e4143.

30. Castellana, S., Vicario, S. and Saccone, C. (2011) Evolutionary patterns of the mitochondrial genome in Metazoa: exploring the role of mutation and selection in mitochondrial protein coding genes. *Genome biology and evolution*, **3**, 1067-1079.

31. Gouy, M., Milleret, F., Mugnier, C., Jacobzone, M. and Gautier, C. (1984) ACNUC: a nucleic acid sequence data base and analysis system. *Nucleic acids research*, **12**, 121-127.

32. Gorman, G.S., Chinnery, P.F., DiMauro, S., Hirano, M., Koga, Y., McFarland, R., Suomalainen, A., Thorburn, D.R., Zeviani, M. and Turnbull, D.M. (2016) Mitochondrial diseases. *Nature reviews. Disease primers*, **2**, 16080.

33. Taylor, R.W., Singh-Kler, R., Hayes, C.M., Smith, P.E. and Turnbull, D.M. (2001) Progressive mitochondrial disease resulting from a novel missense mutation in the mitochondrial DNA ND3 gene. *Annals of neurology*, **50**, 104-107.

34. McFarland, R., Kirby, D.M., Fowler, K.J., Ohtake, A., Ryan, M.T., Amor, D.J., Fletcher, J.M., Dixon, J.W., Collins, F.A., Turnbull, D.M. *et al.* (2004) De novo mutations in the mitochondrial ND3 gene as a cause of infantile mitochondrial encephalopathy and complex I deficiency. *Annals of neurology*, **55**, 58-64.

35. Bugiani, M., Invernizzi, F., Alberio, S., Briem, E., Lamantea, E., Carrara, F., Moroni, I., Farina, L., Spada, M., Donati, M.A. *et al.* (2004) Clinical and molecular findings in children with complex I deficiency. *Biochimica et biophysica acta*, **1659**, 136-147.
