# Supplementary figures and images for: MitImpact 3: modeling the residue interaction network of the Respiratory Chain subunits
Source: Nucleic Acids Res. 2020 Dec 9;49(D1):D1282–8. doi: 10.1093/nar/gkaa1032 (PMC7779045; doi:10.1093/nar/gkaa1032)

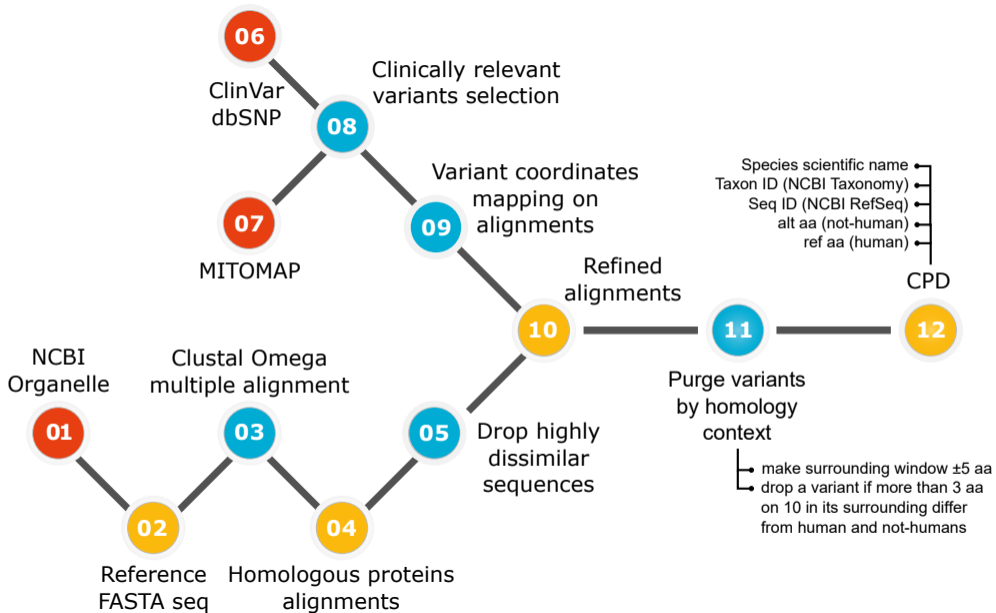

Supplement: gkaa1032_Supplemental_Files [file gkaa1032_supplemental_files.zip › Supplementary_File_1_Figure.pdf]
